# Supplementary material for: Investigations into the In Vitro Metabolism of hGH and IGF-I Employing Stable-Isotope-Labelled Drugs and Monitoring Diagnostic Immonium Ions by High-Resolution/High-Accuracy Mass Spectrometry
Source: Metabolites. 2022 Feb 4;12(2):146. doi: 10.3390/metabo12020146 (PMC8877552; doi:10.3390/metabo12020146)
Supplement: Supplementary file 1 [file metabolites-12-00146-s001.zip › metabolites-1574220-supplementary.pdf]

Investigations into the *in vitro* metabolism of hGH and IGF-I employing stable-isotope labelled drugs and monitoring diagnostic immonium ions by high resolution/high accuracy mass spectrometry

Supplemental Information

Sophia Krombholz [1], Andreas Thomas [1*] and Mario Thevis [1,2]

[1] Institute of Biochemistry/Center for Preventive Doping Research, German Sports University Cologne, Am Sportpark Müngersdorf 6, 50933 Cologne, Germany
[2] European Monitoring Center for Emerging Doping Agents (EuMoCEDA), Cologne/Bonn, Germany

Figure S 1: Product ion mass spectrum of hGH 135-142 (m/z 922.4993); NCE: 25 % 2
Figure S 2: Product ion mass spectrum of hGH 135-143 (m/z 1085.56263); NCE: 26 % 2
Figure S 3: Product ion mass spectrum of hGH 34-44 (m/z 687-3586); NCE: 22 % 2
Figure S 4: Product ion mass spectrum of hGH 125-139 (m/z 832-4147); NCE: 30 % 3
Figure S 5: Product ion mass spectrum of hGH 29-41 (m/z 811-3909); NCE: 25 %; *pE: pyroglutamate 3
Figure S 6: Product ion mass spectrum of hGH 9-16 (m/z 979.5030); NCE: 35 % 3
Figure S 7: Product ion mass spectrum of hGH 86-91 (m/z 771.4036); NCE: 22 % 4
Figure S 8: Product ion mass spectrum of hGH 102-113 (m/z 1302.5848); NCE: 22 % 4
Figure S 9: Product ion mass spectrum of hGH 114-124 (m/z 629.8481); NCE: 22 % 4
Figure S 10: Product ion mass spectrum of hGH 29-38 (m/z 1236.5783); NCE: 25 % 5
Figure S 11: Product ion mass spectrum of hGH 86-92 (m/z 918.4720); NCE: 22 % 5
Figure S 12: Product ion mass spectrum of hGH 1-13 (m/z 745.9037); NCE: 23 % 5
Figure S 13: Product ion mass spectrum of hGH 1-16 (m/z 631.0135); NCE: 22 % 6
Figure S 14: Product ion mass spectrum of IGF 25-36 (m/z 650.8177); NCE: 24 % 6
Figure S 15: Product ion mass spectrum of IGF 25-35 (m/z 572.7671); NCE: 24 % 6
Figure S 16: Product ion mass spectrum of IGF 24-31 (m/z 989.4727); NCE: 25 % 7
Figure S 17: Product ion mass spectrum of IGF 22-36 (m/z 834.3943); NCE: 27 % 7
Figure S 18: Product ion mass spectrum of IGF 4-70 (m/z 921.0660); NCE: 30 % 7
Figure S 19: Product ion mass spectrum of IGF 3-36(m/z 922.1817); NCE: 25 % 8
Figure S 20: Product ion mass spectrum of IGF 3-35 after reduction with TCEP (m/z 1177.8781); NCE: 25 % 8
Figure S 21: Product ion mass spectrum of IGF 3-31 after reduction with TCEP (m/z 1071.8387); NCE: 25 % 8
Figure S 22: Product ion mass spectrum of IGF 3-24 after reduction with TCEP (m/z 1203.55901); NCE: 25 % 9

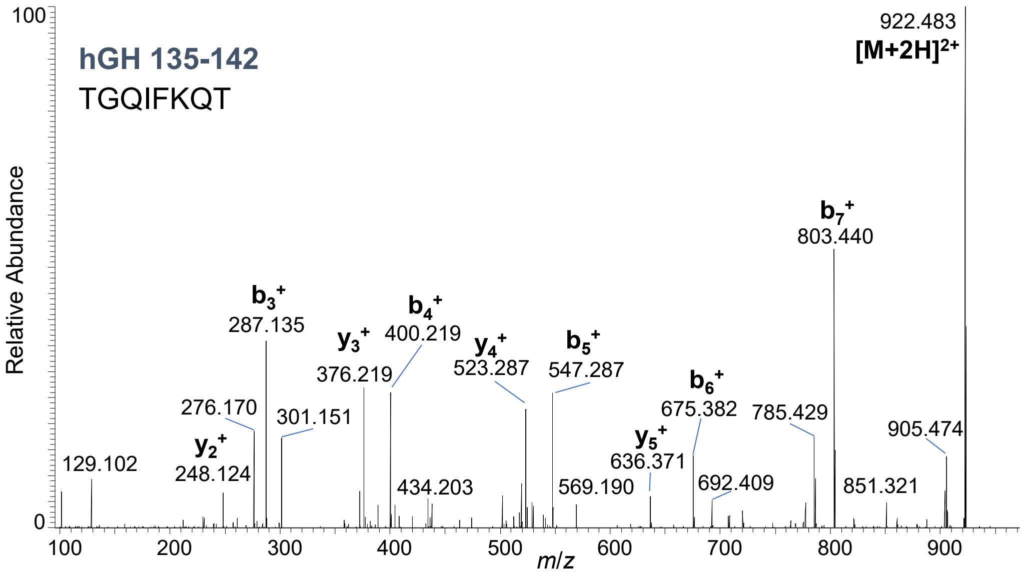

Figure S 1: Product ion mass spectrum of hGH 135-142 (*m/z* 922.4993); NCE: 25 %

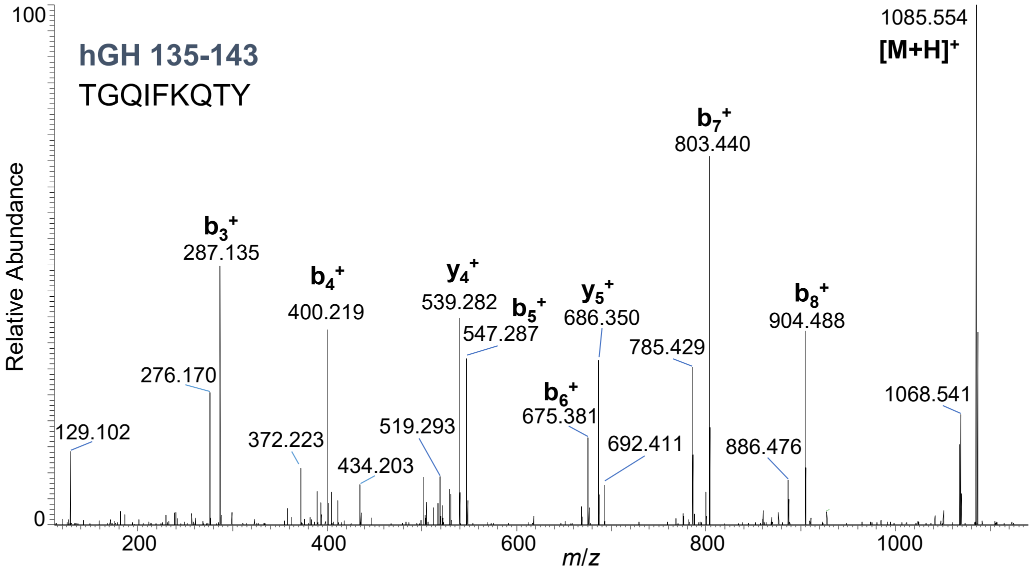

Figure S 2: Product ion mass spectrum of hGH 135-143 (*m/z* 1085.56263); NCE: 26 %

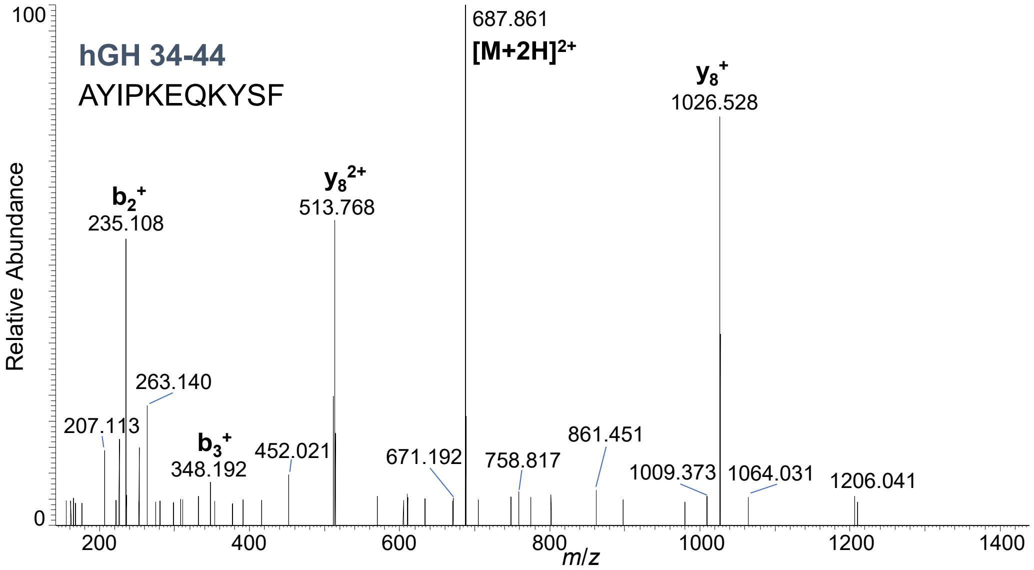

Figure S 3: Product ion mass spectrum of hGH 34-44 (*m/z* 687-3586); NCE: 22 %

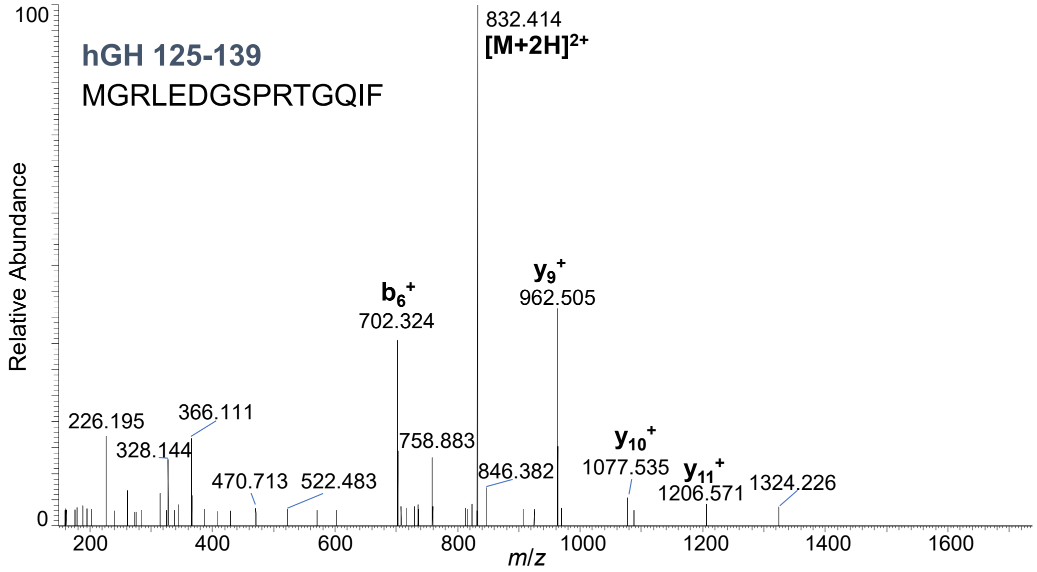

Figure S 4: Product ion mass spectrum of hGH 125-139 (*m/z* 832-4147); NCE: 30 %

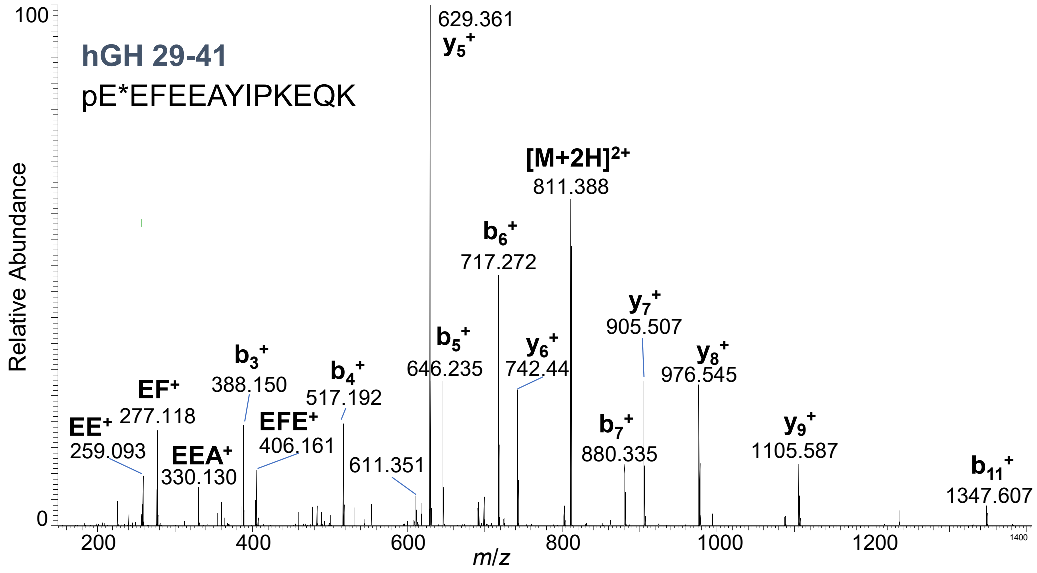

Figure S 5: Product ion mass spectrum of hGH 29-41 (*m/z* 811-3909); NCE: 25 %; *pE: pyroglutamate

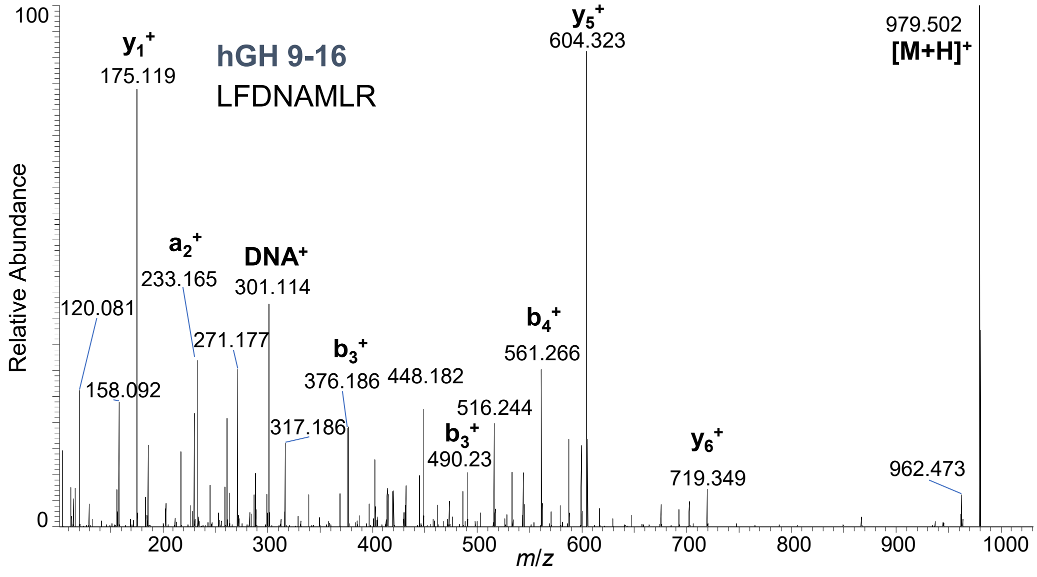

Figure S 6: Product ion mass spectrum of hGH 9-16 (*m/z* 979.5030); NCE: 35 %

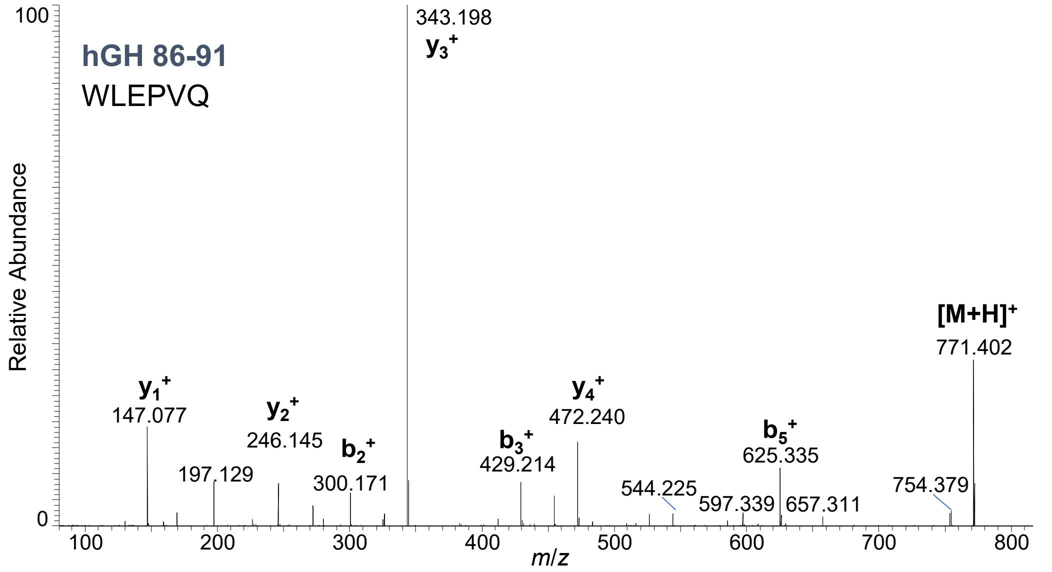

Figure S 7: Product ion mass spectrum of hGH 86-91 (*m/z* 771.4036); NCE: 22 %

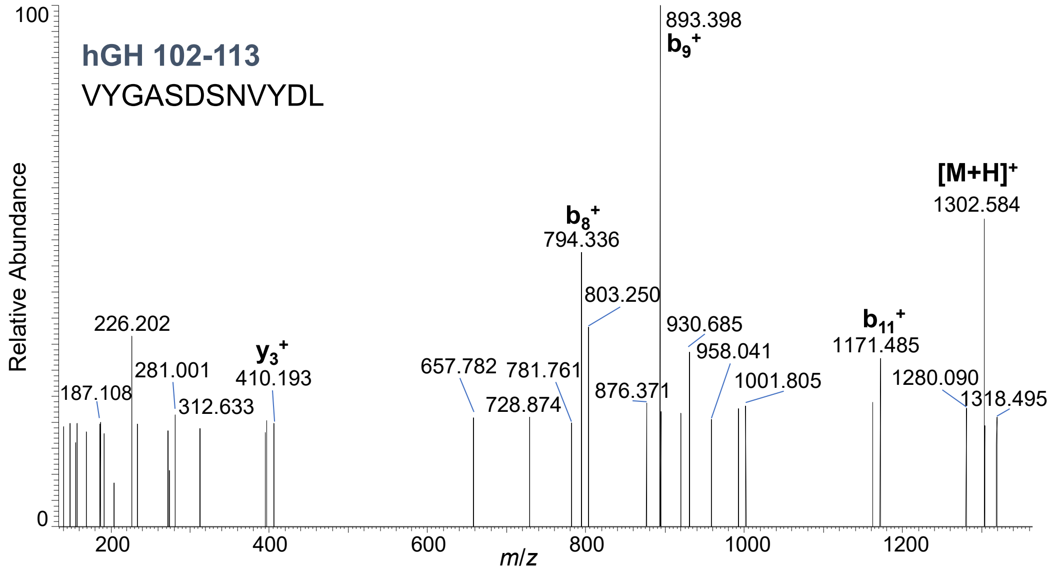

Figure S 8: Product ion mass spectrum of hGH 102-113 (*m/z* 1302.5848); NCE: 22 %

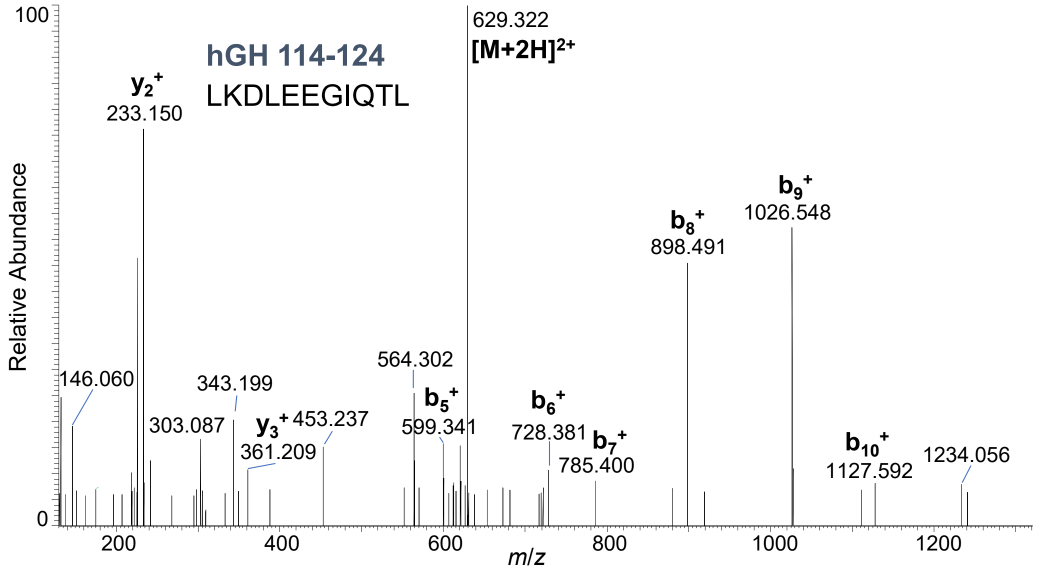

Figure S 9: Product ion mass spectrum of hGH 114-124 (*m/z* 629.8481); NCE: 22 %

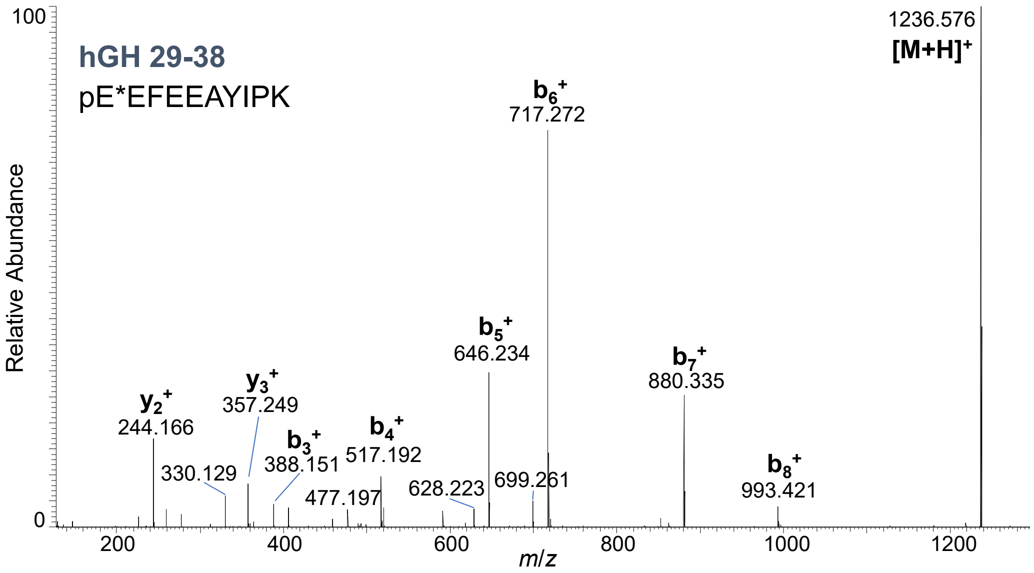

Figure S 10: Product ion mass spectrum of hGH 29-38 (*m/z* 1236.5783); NCE: 25 %

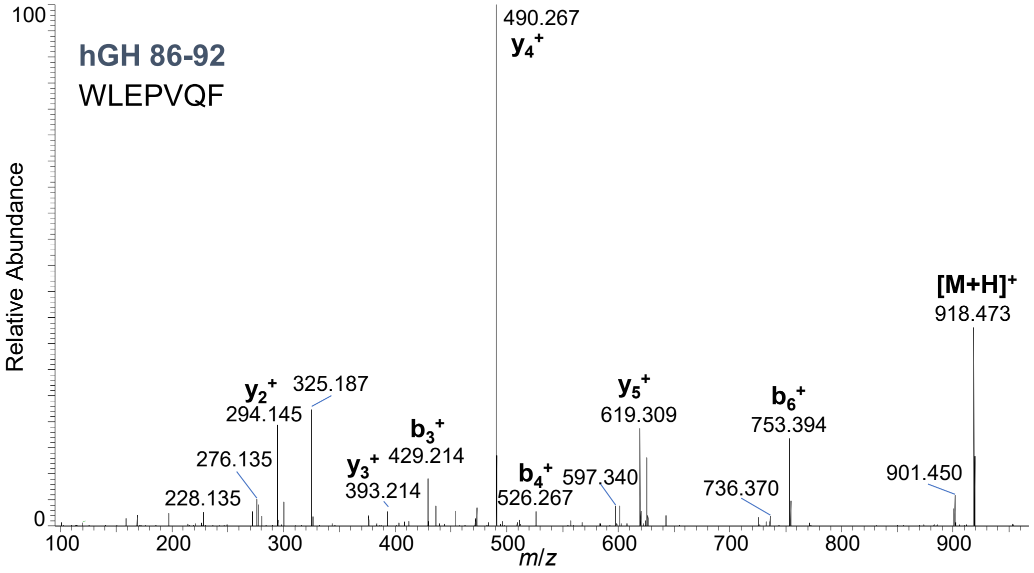

Figure S 11: Product ion mass spectrum of hGH 86-92 (*m/z* 918.4720); NCE: 22 %

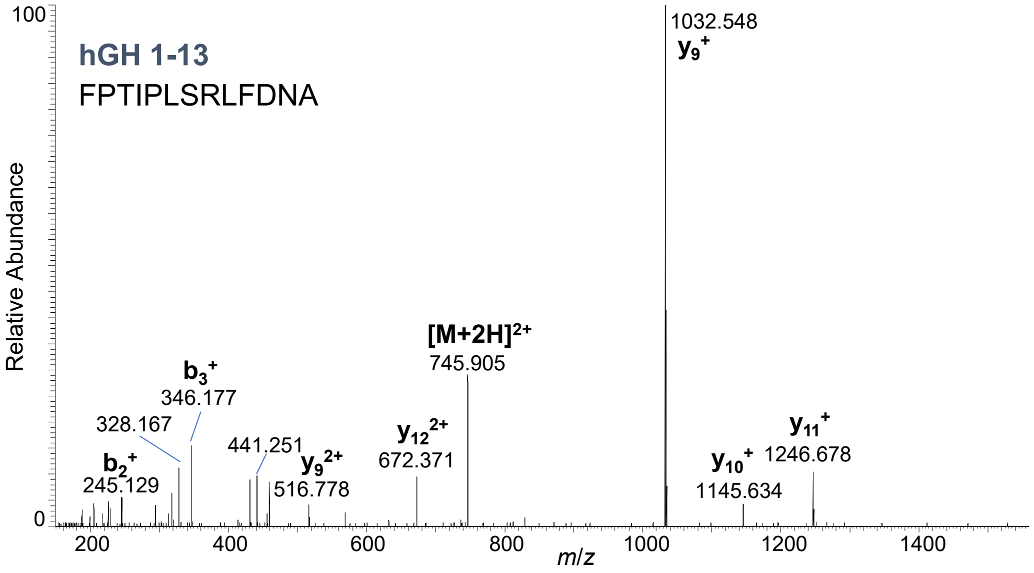

Figure S 12: Product ion mass spectrum of hGH 1-13 (*m/z* 745.9037); NCE: 23 %

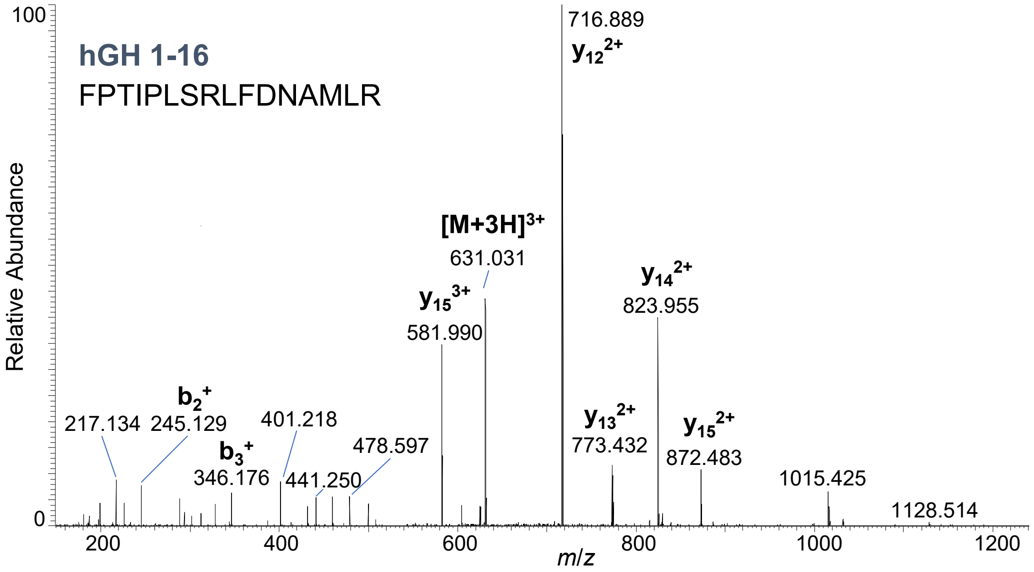

Figure S 13: Product ion mass spectrum of hGH 1-16 (*m/z* 631.0135); NCE: 22 %

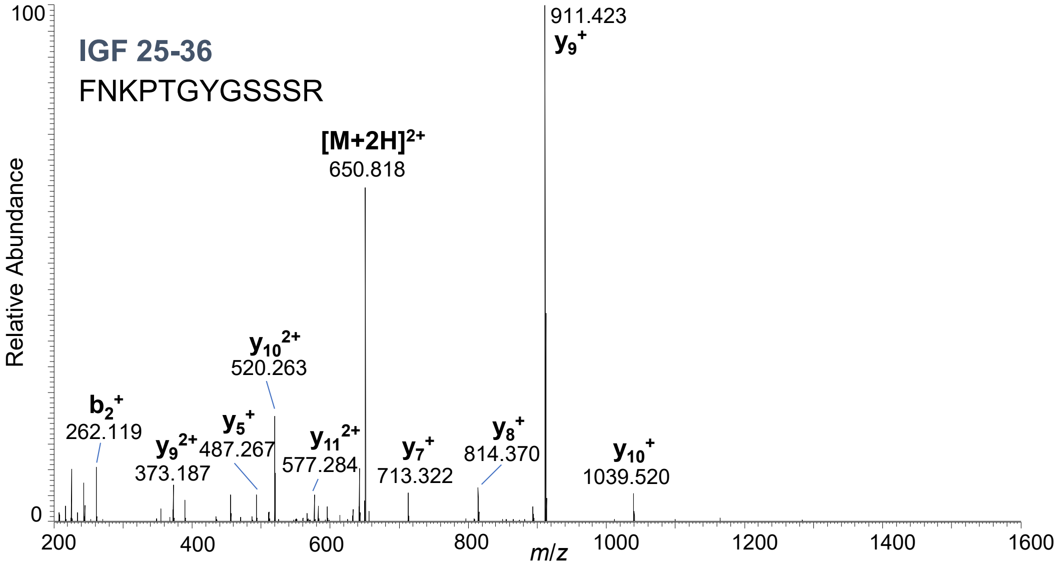

Figure S 14: Product ion mass spectrum of IGF 25-36 (*m/z* 650.8177); NCE: 24 %

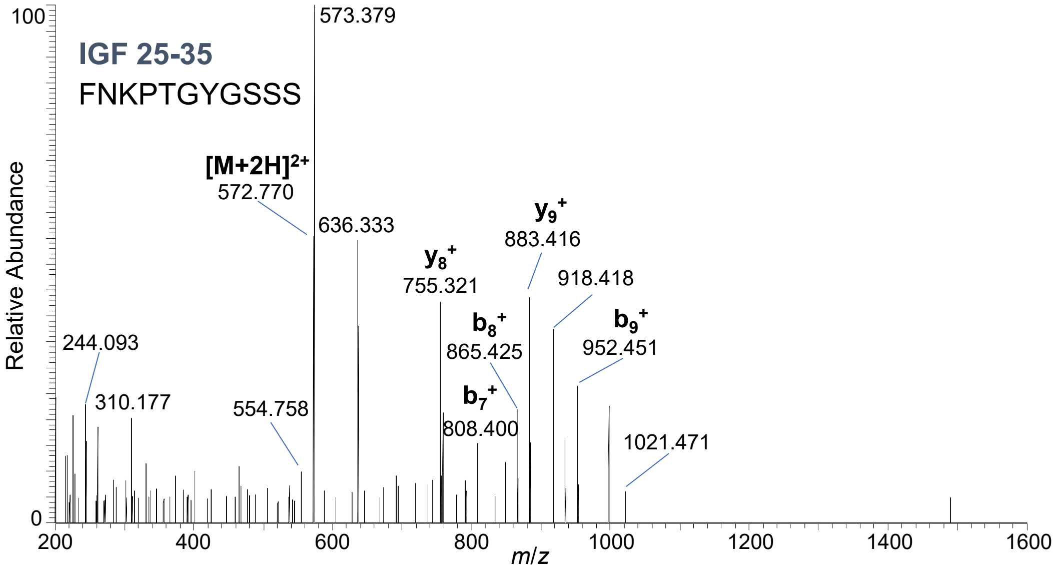

Figure S 15: Product ion mass spectrum of IGF 25-35 (*m/z* 572.7671); NCE: 24 %

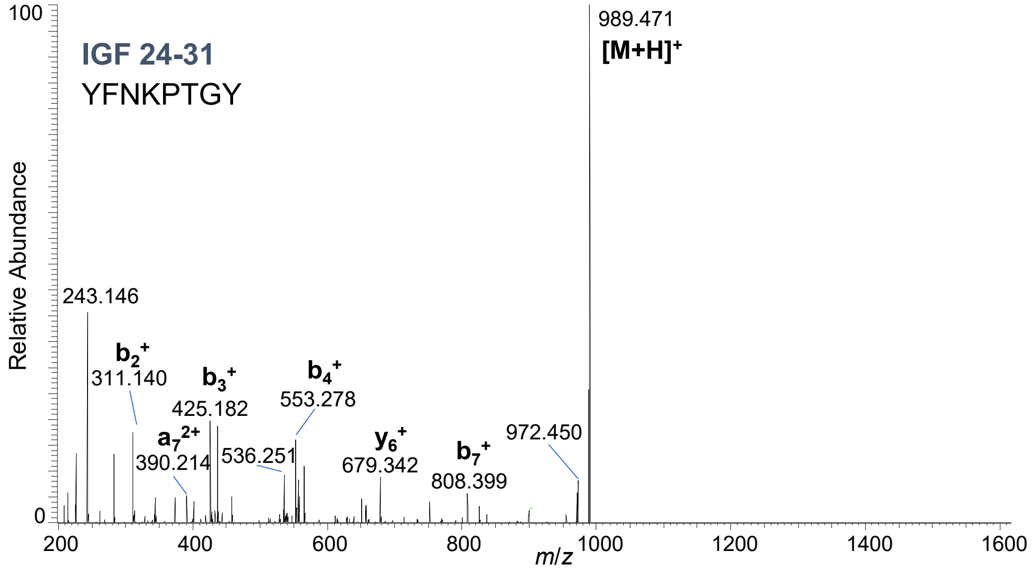

Figure S 16: Product ion mass spectrum of IGF 24-31 (*m/z* 989.4727); NCE: 25 %

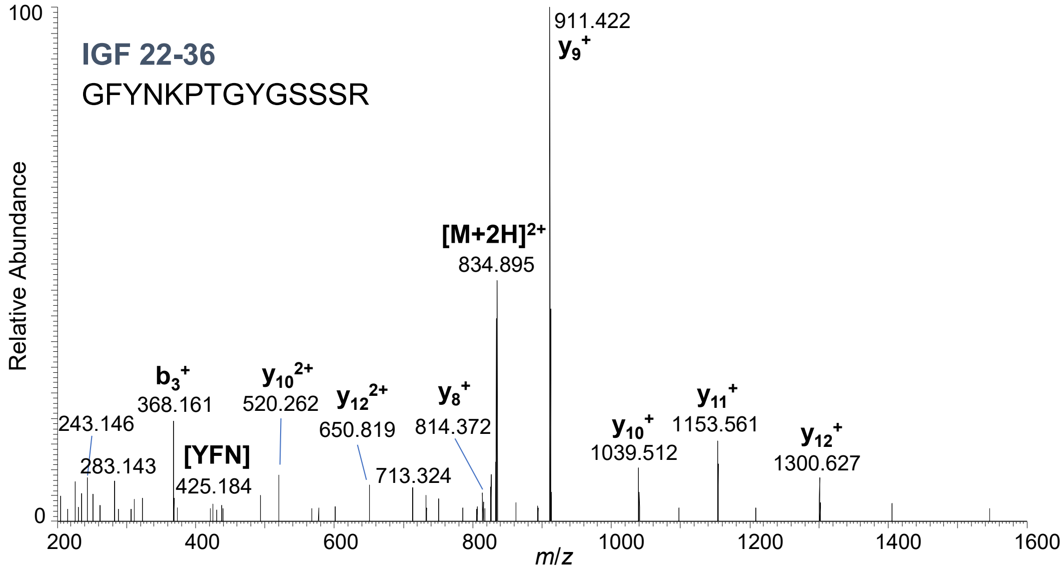

Figure S 17: Product ion mass spectrum of IGF 22-36 (*m/z* 834.3943); NCE: 27 %

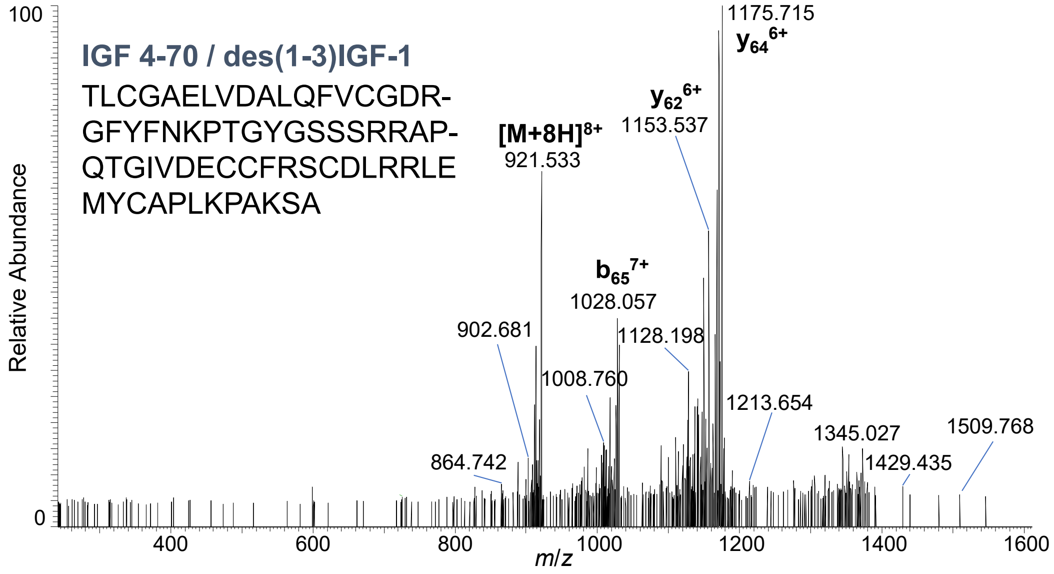

Figure S 18: Product ion mass spectrum of IGF 4-70 (*m/z* 921.0660); NCE: 30 %

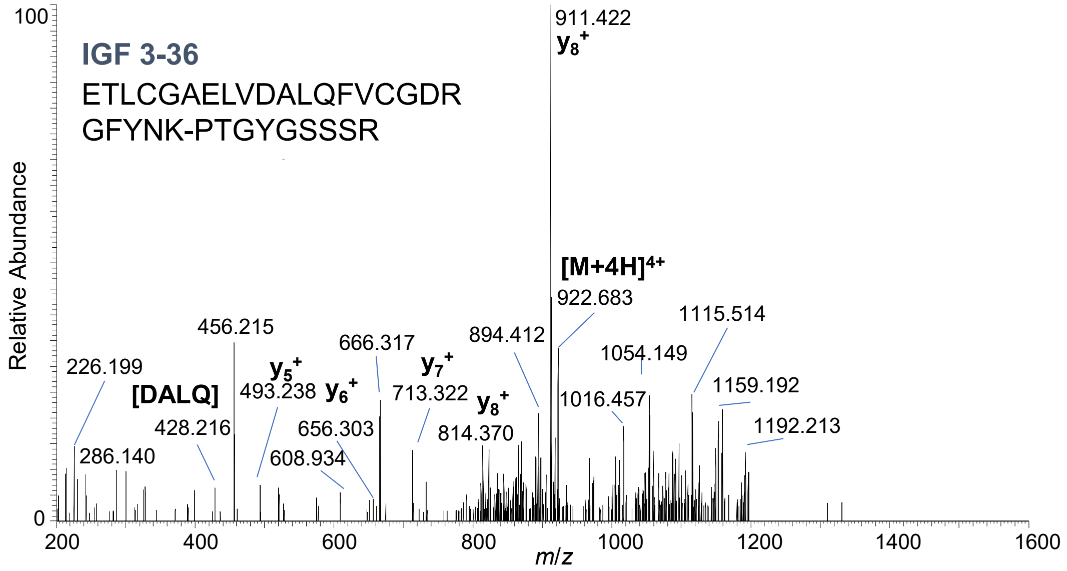

Figure S 19: Product ion mass spectrum of IGF 3-36(m/z 922.1817); NCE: 25 %

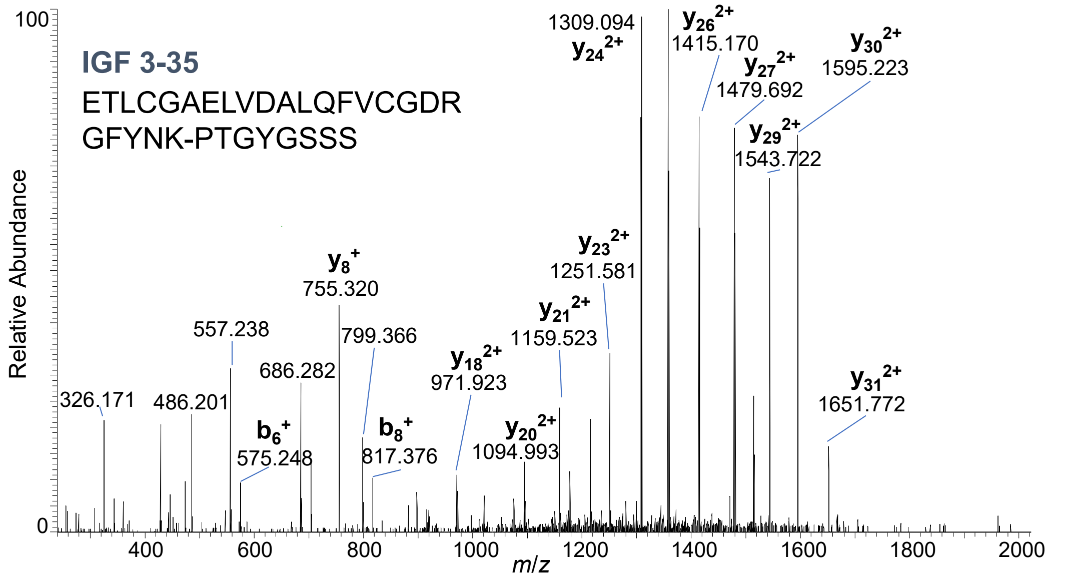

Figure S 20: Product ion mass spectrum of IGF 3-35 after reduction with TCEP (m/z 1177.8781); NCE: 25 %

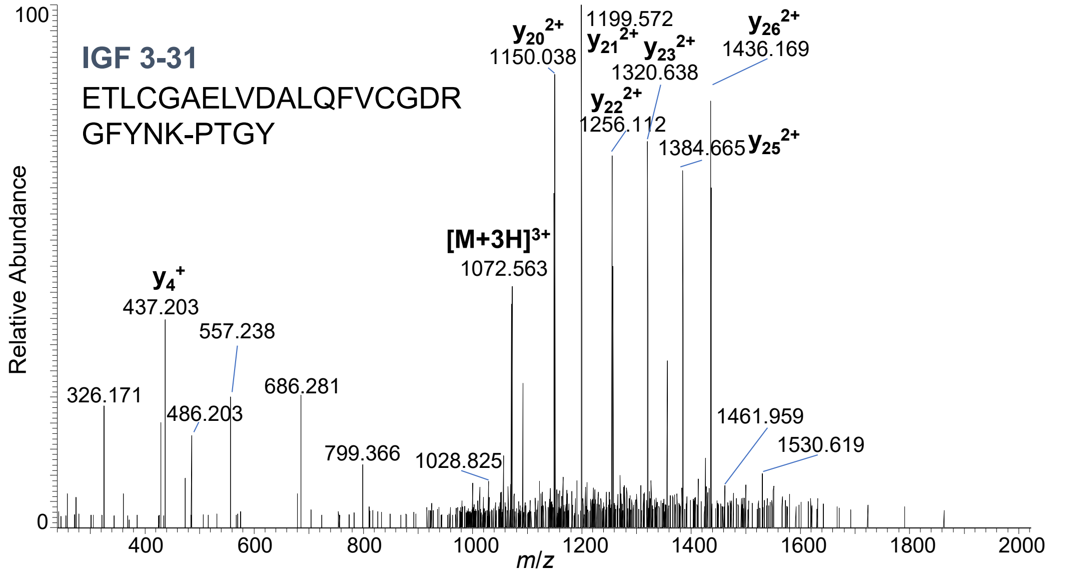

Figure S 21: Product ion mass spectrum of IGF 3-31 after reduction with TCEP (m/z 1071.8387); NCE: 25 %

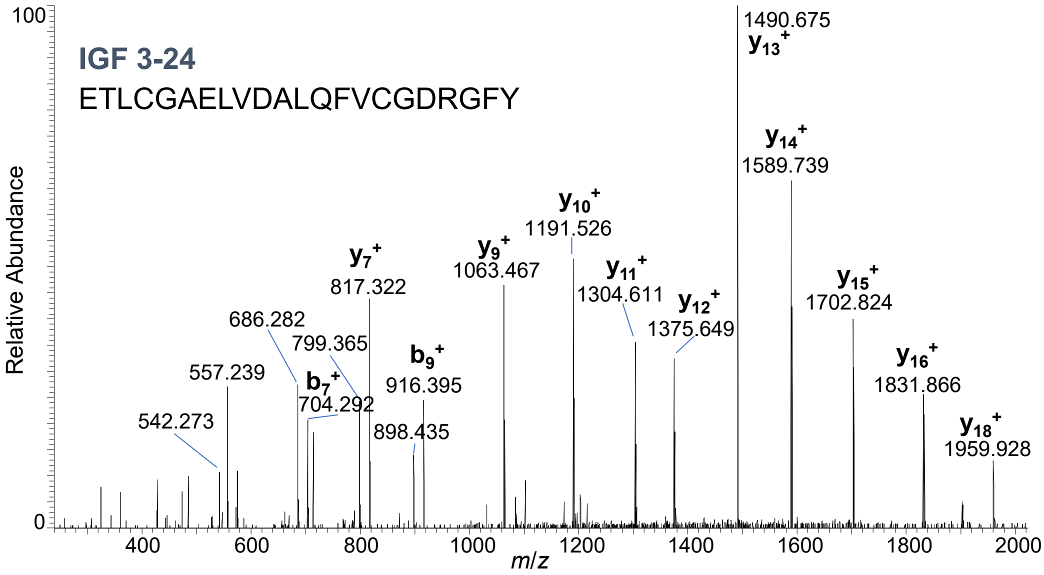

Figure S 22: Product ion mass spectrum of IGF 3-24 after reduction with TCEP (*m/z* 1203.55901); NCE: 25 %
